# Thromboelastometric Profiles of Horses Affected by Exercise-Induced Pulmonary Hemorrhages

**DOI:** 10.4061/2010/945789

**Published:** 2010-09-30

**Authors:** Alessia Giordano, Cecilia Meazza, Marco Salvadori, Saverio Paltrinieri

**Affiliations:** Unit of General Pathology and Parasitology, Department of Veterinary Pathology, Hygiene and Public Health, University of Milan, Via Celoria 10, 20133 Milano, Italy

## Abstract

Exercise-induced pulmonary hemorrhage (EIPH) commonly occurs in race horses. Thromboelastometry (TEM) investigates the whole hemostatic process by evaluating the viscoelastic properties of the blood clot from its formation to fibrinolysis. The aim of this study was to assess whether horses with EIPH have abnormal thromboelastometric profiles. Intrinsic and extrinsic pathways, fibrinogen activity and fibrinolysis were investigated by TEM before and after the race in negative controls and in horses on which EIPH was confirmed by bronchoscopy. Compared with controls, horses with EIPH had an increased coagulability in both pre- and postrace samplings, especially for the intrinsic pathway and for the fibinrolytic activity. These results suggest that coagulation is preactivated in horses prone to develop EIPH, possibly due to recent or recurrent hemorrhage.

## 1. Introduction

Exercise-induced pulmonary haemorrhage (EIPH), with or without evident epistaxis, is frequently observed in race horses and is associated with poor performances [1]. The etiopathogenesis of EIPH is not completely understood. Different pathogenic hypotheses have been proposed for EIPH. Specifically, the possible mechanisms that contribute to determine EIPH in horses include stress failure of pulmonary capillaries, upper airways obstructions, small airways diseases, blood flow redistribution after exercise, altered blood viscosity, and trauma following locomotory impact [1–3]. The possible presence of a coagulopathy, which, however, has not been identified using coagulation times, has also been postulated [4]. 

EIPH is usually diagnosed by clinical examination and by instrumental investigations such as bronchoscopy and/or bronchoalveolar lavage. Both these approaches could be disadvantageous: instrumental procedures are moderately invasive, and the traditional clinical approach, allows to diagnose EIPH only when horses already show evident symptoms (epistaxis, poor performances). The availability of a less invasive technique and/or of a diagnostic test able to early identify this disturbance could be useful to better manage horses with suspected EIPH and/or to prevent possible worsening of the clinical condition.

Thromboelastometry (TEM) investigates the coagulation process by evaluating the viscoelastic properties of the blood clot from its formation to fibrinolysis [5]. It is widely employed in human medicine, and recently it has been validated in many species including equine [6–9]. 

TEM could thus be a useful and noninvasive tool to obtain early information about the haemostatic profile in horses affected by EIPH. 

The hypothesis of this study is that TEM, which provides a more complete overview of the coagulation process (TEM results depend on the interaction between cellular and soluble factors involved in hemostasis) compared with coagulation times (actually based only on the concentration/activity of coagulation factors), can better identify the coagulopathy potentially involved in the pathogenesis of EIPH. The aim of this study is thus to investigate the possible presence of coagulation abnormalities in horses affected by EIPH using TEM.

## 2. Materials and Methods

### 2.1. Animals, Samplings, and Routine Laboratory Tests

All the horses in the present study were from private owners, and both blood withdrawals and bronchoscopies were performed under informed consent and without causing distress to the animals. The inclusion criteria were the absence of previous history of EIPH and/or poor performance for horses belonging to the control group and, conversely, the presence of a previous diagnosis of EIPH or of episodes of poor performance for horses belonging to the EIPH group. The only exclusion criterion, for both groups, was the presence of hematological or biochemical abnormalities potentially influencing the coagulation process (e.g., thrombocytopenia or thrombocytosis, anemia, biochemical changes suggestive of renal failure or of liver damage or insufficiency), as assessed with the panel of tests described below. 

Horses were sampled both before and after a standardized race of 1.600 meters. In all the horses, including those that never had EIPH episodes before this study (control group, see below), one hour after racing, a bronchoscopy using a side-viewing instrument (Side-viewing bronchoscope GF-10, Olympus, Milan, Italy) was done to verify the possible presence of hemorrhages in the respiratory tract. 

Blood samples were collected from the jugular vein, paying attention to avoid unnecessary manipulation of the sampling site, which could result in activation of coagulation. Blood samples were then collected into nonsiliconized vacutainer tubes (Venoject-Serum—code VT-050SU, Terumo Italia Srl, Rome, Italy), to obtain serum for a basic biochemical panel (ALT, ALP, GGT, total protein, albumin, urea, and creatinine) run on an automatic biochemistry analyzer (Cobas Mira, Roche Diagnostic, Basel, Switzerland) using reagents provided by Real Time Diagnostic Systems (Real Time Diagnostic Systems, Viterbo, Italy) and into glass vacutainer tubes specific for coagulation tests (Venoject-Coagulation—code VT-050SBCS, Terumo Italia Srl, Rome, Italy), each containing 0.5 ml buffered sodium citrate (3.8%, corresponding to 0.129 mol/L), which were used to perform routine hematology and thromboelastometry, as described below. The use of sodium citrate, instead of EDTA, is recommended by the manufacturer of the thromboelastometer, since the chelation of calcium induced by this anticoagulant is reversible: this allows the reactivation of coagulation after addition of the calcium chloride included in TEM reagents. The tubes were filled to their maximum capacity (4.5 ml of blood) and transported to the lab within 2 hours at environmental temperature, as recommended to avoid artifactual alteration of thromboelastometric results [8]. Routine hematology was also performed on citrated blood to avoid excessive sampling of blood and because this anticoagulant should be preferred to EDTA for platelet counting in horses [10]. The complete cell blood count was performed using an impedance counter (Hemat 8, SEAC, Calenzano (FI), Italy) already validated for equine blood [11]. Results generated by the instrument were corrected by the dilution factor (1 : 9 v/v) induced by the anticoagulant. On blood smears stained with May Grünwald-Giemsa (Merck, Darmstadt, Germany), the differential leukocyte count was microscopically determined on 100 cells, as well as the platelet estimate, by counting the mean number of platelets in 10 oil immersion fields (magnification: 1000x). The platelet estimate was considered adequate when at least 8 platelets per field were observed.

Based on history and on the results of routine biochemistry, hematology, and bronchoscopy, horses were grouped as follows.

- Negative controls (NC): eleven horses (standardbreds aged 2 to 5 years; 7 females and 4 males), with no history of poor performances and/or of EIPH, which did not show any clinical sign or poor performance during racing. When sampled, these horses did not had clinical or laboratory abnormalities, or bronchoscopic evidence of post-race EIPH.
- EIPH (E): thirteen horses (standardbred horses aged 2 to 5 years; 7 females and 6 males) affected by EIPH. These horses suffered previous episodes of EIPH and, in spite of the lack of clinical and laboratory abnormalities, post-race bronchoscopy revealed the presence of EIPH of grade-1 severity [12].

### 2.2. Thromboelastometric Analyses

Thrombeloastometric analyses were performed using the thromboelastometer Rotem Gamma (TEM international GmbH, Munich, Germany) and reagents provided by the manufacturer of the instrument and previously validated in horses [8]. The following thromboelastometric tests were done: intrinsic pathway (Startem + Intem); extrinsic pathway: (Startem + Extem) fibrinogen activity (Extem + Fibtem); fibrinolysis (Extem + Aptem). The last two tests were performed on 11 out of the 13 samples from horses with EIPH due to technical reasons that hampered a correct evaluation of thromboelastograms regarding fibrinogen activity and fibrinolysis in two samples.

For each test, the following parameters of the thromboelastogram were evaluated.

**Figure 1:**
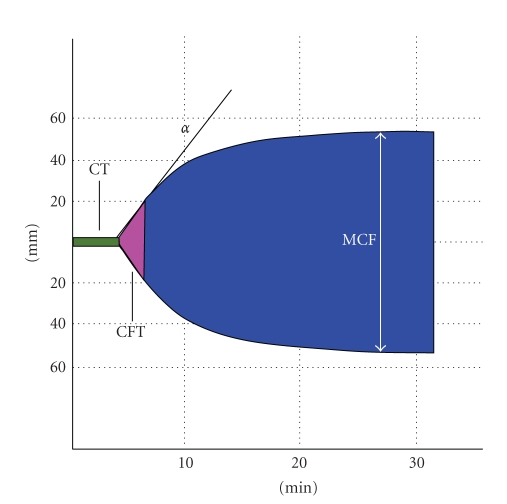
Schematic representation of the thromboelastometric profile. CT: coagulation time (CT); CFT: clot formation time; *α*: alpha-angle; MCF: maximum clot firmness.

- *Coagulation time (CT)* (Figure 1): time (in seconds) between the start of the test and the formation of the first measurable clot (amplitude of the thromboelastogram: 2 mm); the CT basically reflects the activity of plasma coagulation factors.
- *Clot formation time (CFT)* (Figure 1): time (in seconds) necessary to increase the amplitude of the thromboelastometric tracing from 2 to 20 mm; the CFT depends on the initial activation of platelets and fibrinogen.
- *Alpha-angle *(*α*) (Figure 1): slope (expressed in degrees) of the tangent to the elasticity curve; increases or decreases of this angle indicate a trend to hypercoagulation or hypocoagulation, respectively.
- *Maximum clot firmness (MCF)* (Figure 1): maximum strength of the clot (maximum amplitude, in mm, of the thromboelastogram). The MCF depends on both platelet and fibrinogen activation in the presence of factor XIII (which stabilizes the clot).
- *Maximum velocity *(*V*max): maximum rate of clot formation, expressed in mm/min. It is measured on the first derivative generated by the instrument [13] and corresponds to the time required to reach the maximum peak of thrombin generation.
- *Area under the curve (AUC)*: the area under the velocity curve measured on the first derivative, expressed in mm^2^. The AUC equals the endpoint minus the starting point of the elasticity curve.

Theoretically, conclusive information about fibrinolysis can be achieved only after 60 minutes, when lysis is complete and an additional parameter (maximum lysis or ML) is provided by the instrument. From a practical point of view, however, when fibrinolysis is activated, the MCF begins to decrease earlier and at 30 mins the instrument allows the operator to detect an increased rate of lysis (LY30). In this study increases of LY30 were never recorded (data not shown) and thromboelastometric runs were thus stopped 30 mins after the end of the CFT.

### 2.3. Statistical Analysis

Mean values and standard deviation were calculated for each group before and after the race. All the statistical analyses were performed in an Excel (Microsoft Corp, Redmond, WA, USA) spreadsheet with the Analyse-it set of macroinstructions (Analyse-it Software Ltd, Leeds, UK). A Kolmogorov Smirnov test was used to determine the data distribution. Results obtained before and after racing were compared to each other using a Wilcoxon paired *t*-test. A Mann-Withney *U* test was employed to compare to each other the results from group NC and E either pre-race or post-race. Differences were considered significant at *P* < .05.

## 3. Results

### 3.1. Hematology

None of the horses enrolled in the study had signs of hepatic or renal failure (data not shown) or hematological changes potentially influencing the coagulation process (Table 1). 

Prerace RBCs were significantly higher in controls than in horses with EIPH. By contrast, no significant differences were found between the hematocrit (Hct) of controls and of horses with EIPH. None of the horses was anemic or had RBC or Hct values higher than the reference range of the laboratory.

After the race, a significant increase of RBCs and Hct, compared with pre-race values, both in controls and in horses with EIPH was detected. Also after the race, however, none of the horses had RBC or Hct values higher than the reference range, indicating that exercise induced only a “relative hemoconcentration” (i.e., an increase of Hct or RBC numbers compared with pre-race values) and not a “true hemoconcentration” (i.e., an increase of Hct or RBC numbers up to values higher than the upper reference limit). No differences between RBC or Hct values recorded in controls and in horses with EIPH were detected after the race. 

No significant differences between the groups were found in pre-race WBC or platelet counts. In both the groups, post-race samples were not significantly different compared with the pre-race ones. After the race, the platelet number of horses with EIPH was significantly higher than that of controls.

### 3.2. Thromboelastometric Results

Thromboelastometric results are summarized in Tables 2 (pre-race) and 3 (post-race). 

Compared with controls, in EIPH-affected horses the activation of clotting was more rapid than in controls (shorter CT and CFT), as well as the whole coagulation process (higher *V*max and AUC), and the clot was stronger (higher MCF and *α* angle) both before and after racing. This trend was evident in all the test but it was often non statistically significant, likely due to a high individual variability. Significant differences were recorded for the intrinsic pathway (CFT, *α* angle and *V*max, in both pre- and post-race samples) for fibrinolysis (MCF and AUC in both pre- and post-race samples) and for the extrinsic pathway (CT in the post-race sample). 

As a general trend, in both controls and EIPH-affected horses after racing the coagulability decreased (longer CT and CFT and reduced MCF). Specifically, the differences between pre- and post-race results were rarely significant only for the extrinsic pathway (increased CFT and decreased AUC for both groups, lower *α* angle and MCF for controls) and the intrinsic pathway (longer CFT and reduced MCF and AUC for horses with EIPH) for fibrinogen activity (reduced MCF, *V*max, and AUC for both groups) and for fibrinolysis (reduced MCF and *V*max in horses with EIPH).

## 4. Discussion

Both hematological and tromboelastometric results recorded in controls either before or after the race were similar to those obtained in a previous study [8]. Basically, these results confirm that exercise induces a moderate relative hemoconcentration and reduces the coagulability in healthy animals. 

Compared with controls, EIPH-affected horses showed a trend to a hypercoagulable state (shorter times of clot activation and higher clot firmness for most of the considered tests) before the race, when, however, many differences were not statistically significant, likely due to the individual variability. In a previous study it has been demonstrated that hypercoagulability can depend on a decreased RBC mass [8] but it is unlikely that this occurred in EIPH horses before the race since, in spite of a lower number of RBCs, the erythroid mass was not decreased, as demonstrated by the lack of significant differences of Hct. Similarly, the hypercoagulability recorded in horses with EIPH before the race does not depend on a different platelet number, since no significant differences regarding platelet counts of the two groups were recorded. Theoretically, the increased coagulability recorded after the race could depend on hyperfibrinogenemia. The concentration of fibrinogen has not been measured using standard methods but in previous studies it has been demonstrated that the concentration of fibrinogen correlates well with the thromboelastometric evaluation of fibrinogen [5]. It is thus unlikely that horses included in this study were hyperfibrinogenemic either before or after the race since in both samplings the activity of fibrinogen was comparable with that previously recorded in healthy horses [8].

After the race coagulability decreased (thromboelastograms characterised by longer CT and CFT and less effective clot firmness) also in horses affected by EIPH, as in controls, probably due to the hemoconcentration recorded in both groups, since, as previously mentioned, an inverse correlation between the erythroid mass and most TEM parameters has been demonstrated [8]. The immediate consequence of the parallel behaviour of TEM parameters in the two groups is that also after the race EIPH horses have a “hypercoagulable state” compared with controls, and, also in this case, this phenomenon cannot depend on differences of Hct or RBC counts, since these parameters were not significantly different in EIPH groups compared with controls. Conversely, the hypercoagulability could be associated with the high platelet count recorded after the race in EIPH horses.

These results suggest that in EIPH-affected horses hemostasis is activated in spite of the lack of differences in prothrombin time and in activated thromboplastin time demonstrated by others [4]. 

The results of the present study do not allow to understand the cause and/or the mechanism responsible for the trend to hypercoagulability recorded in horses with EIPH. All the hemostatic pathways, including fibrinolysis, showed the same trend, although with different intensity. It is thus unlikely that the increased coagulability depends on a primary activation of a specific step of the hemostasis. Rather, previous studies demonstrated that an increased coagulability can be detected by thromboelastometry when coagulation is activated by spontaneous or surgical bleeding [5]. It is possible that TEM results in EIPH horses depend on recent hemorrhagic episodes occurred before the pre-race sampling or on recurrent hemorrhage which could “chronically” activate coagulation. If this interpretation is correct, the mechanism responsible for the hemorrhage remains to be elucidated. All the hypotheses raised in the past about the pathogenesis of EIPH [1–3] can be possibly involved in the damage of vessel walls. Further studies should be designed in the future to assess whether the hypercoagulability detected before the race is only a consequence of hemorrhage or if it could work as a predisposing factor for EIPH.

## 5. Conclusions

The results of this study provide useful additional information to the understanding of the pathogenesis of EIPH in horses: specifically, TEM analysis confirmed that EIPH is not associated with decreased blood coagulability. On the contrary, horses affected by EIPH tend to be hypercoagulable before the race, possibly due to pre-existing hemorrhage.

## Figures and Tables

**Table 1:** Mean values ± standard deviation (median values) recorded before the race.

	Controls		EIPH	
	Prerace	Postrace	Prerace	Postrace
RBC ×10^6^/*μ*l	8.7 ± 0.6(8.7)	9.6 ± 0.8(9.6)^†^	8.0 ± 0.5(7.8)*	8.9 ± 1.1(8.8)^†^
Hct (%)	32.9 ± 6.2(34.9)	38.6 ± 2.6(38.9)^†^	32.8 ± 1.4(32.2)	37.1 ± 4.4(36.3)^†^
WBC ×10^3^/*μ*l	8.5 ± 1.7(8.4)	8.9 ± 2.2(8.9)	8.2 ± 1.8(7.9)	8.2 ± 1.6(7.8)
Platelets ×10^3^/*μ*l	143.8 ± 36.5(135.0)	145.9 ± 29.5(150.0)	144.3 ± 49.3(134.0)	188.1 ± 39.4(181.0)*

*P < .05versus results of controls in the same type of sampling (e.g., pre-race or post-race); ^†^ *P* < .05 versus pre-race results of the same group (e.g., controls or EIPH).

**Table 2:** Mean values ± standard deviation (median values) recorded before the race.

		CT (sec)	CFT (sec)	MCF (mm)	*α* (°)	*V*max (mm/min)	AUC (mm^2^)
EXT	NC	56.7 ± 14.6	140.±19.3	54.4 ± 5.0	68.0 ± 5.4	12.3 ± 2.5	5420.1 ± 498.6
		(59.0)	(140.0)	(54.0)	(70.0)	(11.0)	(5358.0)
	E	49.9 ± 4.6	122.7 ± 24.1	57.8 ± 4.5	71.7 ± 5.0	14.7 ± 4.1	5754.0 ± 411.3
		(48.4)	(127.8)	(57.6)	(70.5)	(14.1)	(5739.3)
INT	NC	277.0 ± 37.5	124.1 ± 22.5	47.0 ± 5.8	66.1 ± 3.7	12.1 ± 6.0	4739.0 ± 587.8
		(287.0)	(124.0)	(45.0)	(66.0)	(10.0)	(4595.0)
	E	291.1 ± 33.5	100.6 ± 12.5	50.3 ± 4.4	69.9 ± 1.7	14.0 ± 3.9	4965.4 ± 418.2
		(294.3)	(102.2)	(49.8)	(69.8)	(12.8)	(4871.2)
FIB**^†^**	NC	51.0 ± 4.5	Nd	15.3 ± 3.3	70.7 ± 6.9	14.6 ± 2.7	1504.0 ± 329.2
		(51.0)		(15.0)	(72.0)	(14.0)	(1485.0)
	E	47.4 ± 3.2	Nd	16.9 ± 3.4	75.2 ± 2.7	17.0 ± 3.6	1673.6 ± 368.5
		(47.5)		(16.5)	(74.0)	(16.0)	(1623.0)
LYS**^†^**	NC	50.5 ± 3.4	162.0 ± 22.5	48.7 ± 3.1	65.8 ± 7.0	11.2 ± 2.6	4825.8 ± 297.6
		(50.5)	(165.5)	(48.5)	(67.5)	(12.0)	(4783.5)
	E	52.6 ± 5.7	142.9 ± 31.6	53.8 ± 4.2	67.3 ± 7.7	14.0 ± 3.8	5345.4 ± 483.0
		(53.5)	(140.0)	(53.5)	(66.0)	(13.5)	(5305.0)

NC: negative controls; E: EIPH; EXT: extrinsic pathway; INT: intrinsic pathway; FIB: fibrinogen activity; LYS: fibrinolysis; nd: not determinable; in bold *P* < .05 versus the pre-race NC value; **^†^** Fibrinogen and fibrinolytic activities were determined in only 11 samples.

**Table 3:** Mean values ± standard deviation (median values) recorded after the race.

		CT (sec)	CFT (sec)	MCF (mm)	*α* (°)	*V*max (mm/min)	AUC (mm^2^)
EXT	NC	58.4 ± 10.4	150.4 ± 29.6	51.4 ± 5.4	63.8 ± 6.1	11.0 ± 2.3	5126.6 ± 511.0
		(57.0)	(148.0)*	(53.0)*	(66.0)*	(11.0)	(5252.0)*
	E	51.3 ± 7.9	137.8 ± 28.5	56.8 ± 5.4	66.8 ± 6.5	11.3 ± 2.1	5324.1 ± 367.5
		(51.7)	(136.1)*	(56.1)	(67.2)	(11.9)	(5343.0)*
INT	NC	257.±45.66	131.0 ± 28.3	45.0 ± 5.1	66.2 ± 4.0	11.0 ± 2.2	4527.8 ± 501.3
		(234.0)	(135.0)	(43.0)	(66.0)	(10.0)	(4318.0)
	E	262.6 ± 42.1	101.9 ± 15.4	49.8 ± 5.4	72.0 ± 3.5	14.0 ± 2.1	5002.2 ± 415.0
		(247.9)	(100.6)*	(49.9)*	(71.0)	(14.1)	(4951.2)*
FIB**^†^**	NC	52.0 ± 6.2	Nd	14.0 ± 3.6	68.0 ± 4.2	11.4 ± 2.9	1341.3 ± 348.2
		(54.0)		(14.0)*	(68.0)	(12.0)*	(1328.0)*
	E	48.2 ± 6.2	Nd	14.1 ± 3.9	70.9 ± 8.8	13.9 ± 5.1	1376.0 ± 414.7
		(48.0)		(15.0)*	(75.0)	(14.0)*	(1398.0)*
LYS**^†^**	NC	53.5 ± 5.9	180.5 ± 23.7	46.0 ± 3.0	61.0 ± 7.1	10.0 ± 2.4	4575.2 ± 269.7
		(53.5)	(183.0)	(45.0)	(60.5)	(10.5)	(4511.5)
	E	47.3 ± 5.3	144.7 ± 31.3	52.2 ± 4.8	66.5 ± 6.3	12.2 ± 2.5	5278.5 ± 475.5
		(47.5)	(144.0)	(52.0)*	(67.0)	(12.0)*	(5186.5)

NC: negative controls; E: EIPH; EXT: extrinsic pathway; INT: intrinsic pathway; FIB: fibrinogen activity; LYS: fibrinolysis; nd: not determinable; in bold: *P* < .05 versus the post-race NC value; *P < .05 versus the corresponding pre-race value of the same group; **^†^**Fibrinogen and fibrinolytic activities were determined in only 11 samples.
